# Characterizing the DNA Damage Response by Cell Tracking Algorithms and Cell Features Classification Using High-Content Time-Lapse Analysis

**DOI:** 10.1371/journal.pone.0129438

**Published:** 2015-06-24

**Authors:** Walter Georgescu, Alma Osseiran, Maria Rojec, Yueyong Liu, Maxime Bombrun, Jonathan Tang, Sylvain V. Costes

**Affiliations:** 1 Lawrence Berkeley National Laboratory, Life Sciences Division, Berkeley, CA, United States of America; 2 Department of Pathology, Beth Israel Deaconess Medical Center, Harvard Medical School, Boston, MA, 02215, United States of America; 3 CNRS, UMR 6158, LIMOS, F-63173, Aubiere, France; The University of Hong Kong, HONG KONG

## Abstract

Traditionally, the kinetics of DNA repair have been estimated using immunocytochemistry by labeling proteins involved in the DNA damage response (DDR) with fluorescent markers in a fixed cell assay. However, detailed knowledge of DDR dynamics across multiple cell generations cannot be obtained using a limited number of fixed cell time-points. Here we report on the dynamics of 53BP1 radiation induced foci (RIF) across multiple cell generations using live cell imaging of non-malignant human mammary epithelial cells (MCF10A) expressing histone H2B-GFP and the DNA repair protein 53BP1-mCherry. Using automatic extraction of RIF imaging features and linear programming techniques, we were able to characterize detailed RIF kinetics for 24 hours before and 24 hours after exposure to low and high doses of ionizing radiation. High-content-analysis at the single cell level over hundreds of cells allows us to quantify precisely the dose dependence of 53BP1 protein production, RIF nuclear localization and RIF movement after exposure to X-ray. Using elastic registration techniques based on the nuclear pattern of individual cells, we could describe the motion of individual RIF precisely within the nucleus. We show that DNA repair occurs in a limited number of large domains, within which multiple small RIFs form, merge and/or resolve with random motion following normal diffusion law. Large foci formation is shown to be mainly happening through the merging of smaller RIF rather than through growth of an individual focus. We estimate repair domain sizes of 7.5 to 11 µm^2^ with a maximum number of ~15 domains per MCF10A cell. This work also highlights DDR which are specific to doses larger than 1 Gy such as rapid 53BP1 protein increase in the nucleus and foci diffusion rates that are significantly faster than for spontaneous foci movement. We hypothesize that RIF merging reflects a "stressed" DNA repair process that has been taken outside physiological conditions when too many DSB occur at once. High doses of ionizing radiation lead to RIF merging into repair domains which in turn increases DSB proximity and misrepair. Such finding may therefore be critical to explain the supralinear dose dependence for chromosomal rearrangement and cell death measured after exposure to ionizing radiation.

## Introduction

Double strand breaks (DSB) are the most deleterious type of DNA damage. Even a single DSB can kill a cell or even worse lead to genomic instability, which in turn has been shown to contribute to cancer progression [1]. In recent years, the use of repair proteins tagged with fluorescent markers has greatly expanded our knowledge of the dynamics and mechanisms of DSB repair. The presence of DNA repair centers formed by mobilization and clustering of multiple DSBs has been hypothesized by Savage et al [2] and further investigated by our group [3, 4]. DSBs are clearly mobile and are preferentially repaired in specific regions of the nucleus in budding yeast and Drosophila [5–7]. However in mammalian cells some studies claim that repair sites are fixed [8–10] while others report that DSB repair sites do explore the nuclear domain [11, 12]; We previously characterized the kinetic properties of 53BP1-GFP proteins that form RIFs after exposure to high and low-LET [4]. In this later approach, a mathematical model interpolating RIF resolution kinetics suggested that DSBs move and cluster at common repair sites in mammalian cells. Simulations of DSBs constrained within repair domains have recently been shown to explain the strong downward curvature observed in the low survival of human breast cells exposed to high doses of radiation [13]. However, there has not been definite biological evidence of repair domains in human cells. In the present study, we use time-lapse movies of RIF movement along with cell tracking, linear optimization and feature classification algorithms to characterize RIF formation, and RIF mobility, bringing the missing biological evidence for DNA repair domains in human cells.

## Materials and Methods

### Cell culture

Nonmalignant human mammary epithelial cell line MCF10A were grown in DMEM-F12 supplemented with 5% horse serum, 0.02ug/ml of EGF, 10ug/ml of insulin, 0.5ug/ml of hydrocortisone, 0.1ug/ml of cholera toxin, and 1X penicillin/streptomycin. Cells were incubated at 37ºC and 5% CO2.

### Generation of stable MCF10A 53BP1-mCherry/H2B-GFP

**Fig 1:**
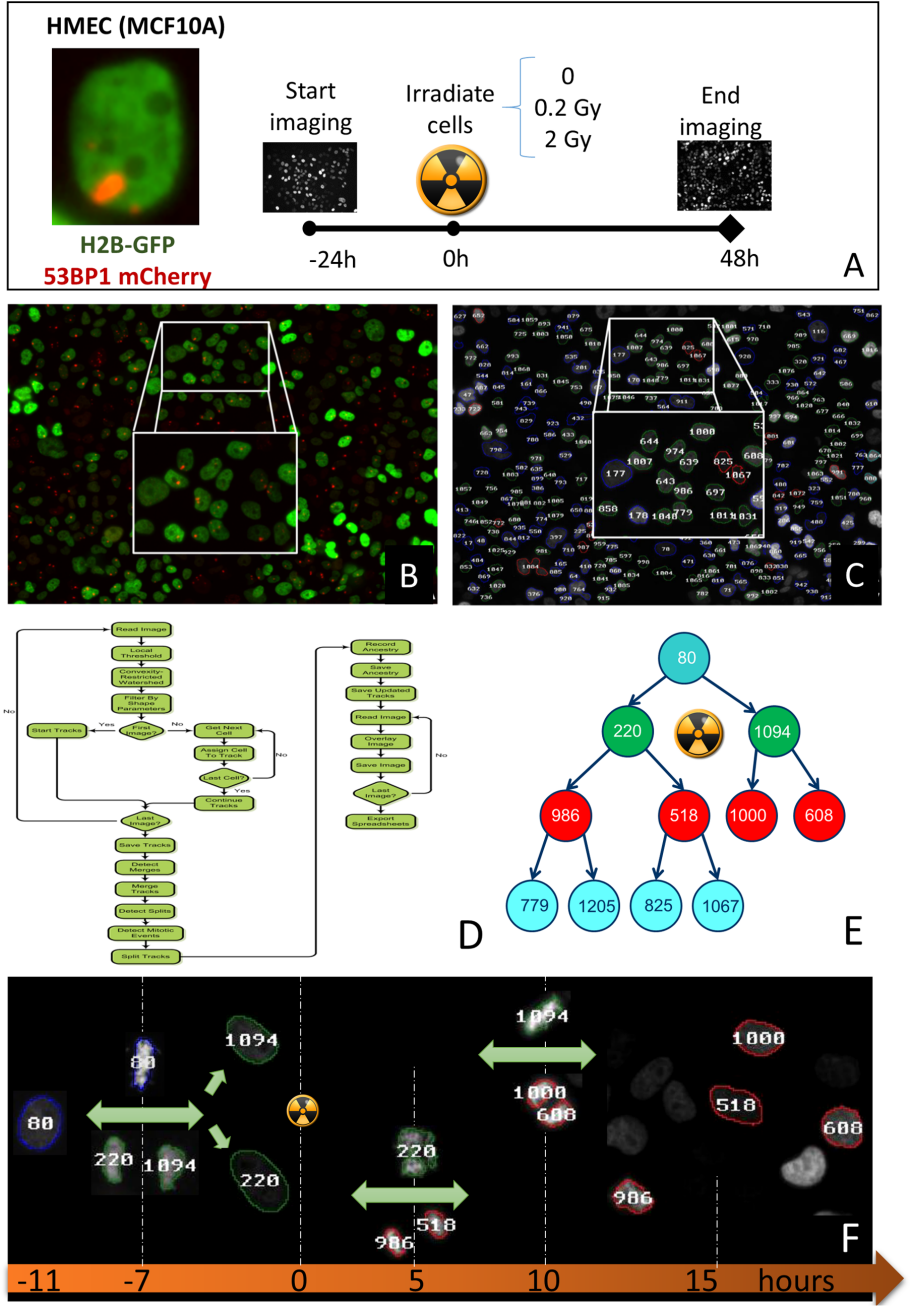
Experimental Setup. (A) Nonmalignant human mammary cells (MCF10A) were infected with two lentiviruses encoding for 53BP1-mCherry and H2B-GFP. Image on left shows a green nucleus and a large focus in a non-irradiated cell. These large spontaneous foci are commonly observed in MCF10A. A stable line was obtained by sorting cells with FACS. Time-lapse experiments were performed with cells being imaged at least 24 hours before ionizing radiation and 24 hours post-irradiation. (B) Time-lapse frame showing cell nuclei (green channel) and 53BP1 spots in the nucleus 24 hours post-irradiation with a 0.2 Gy dose. (C) Same frame as in B after time-lapse series has been processed. Cell outlines indicate cell generation since beginning of movie acquisition (blue = 1,green = 2, red = 3), numbers indicate cell ID. (D) Cell tracking and mitotic event detection flowchart. (E) Typical ancestry tree generated after image processing. Cell 80 detected in the first frame splits into daughter cells 220 and 1094 which are irradiated. Subsequent daughter cells are also tracked up to the fourth generation cells 779, 1205, 825 and 1067. (F) Image montage showing mitotic events for the cells in the ancestry tree in (E).

MCF10A were transduced with Lipofectamine to express histone H2B-GFP plasmid (Fig 1). Lentivirus expressing 53BP1-mCherry was then used to infect cells. Cells were monitored for 24 hours before and after exposures as depicted in Fig 1A.

### Live cell setup

Microscope was fully enclosed inside a lead incubator designed in-house to block radiation from leaking out. When monitoring the number of foci/cell as a function of time, cells had significant amounts of background 53BP1 foci at the beginning of the experiment. We assume this was the result of trypsinization happening 24 hours before starting imaging, oxidative stress and inadequate pH due to temporary lack of CO_2_ as cells went from being plated to being setup on our microscope. We monitored by time-lapse imaging cells for 24 hours before irradiation, letting them equilibrate in their new environment. During equilibration, spontaneous foci/cell rapidly decreased to 5 foci/cell as previously reported by immunocytochemistry [14].

### Radiation Protocol

Cells were irradiated without removal from the microscope incubation hood using a custom-mounted MiniX 40kV X-ray tube. Beam was placed 1 cm above 8 well coverglass (Nunc, Lab-Tek) with a brass collimator leading to a beam size of 5 mm. Different radiation doses ranging from a maximum of 4 Gy to a minimum of 0.1 Gy were delivered at a dose-rate of 1.2 Gy/min. Dose rate when radiation hits the media in each well was 2.08 Gy/min and was attenuated to 1.2 Gy/min when slides were placed in the beam with 400 **μ**l of media in each well. All dose measurements were done using Gafchromic films EBT3 (Ashland Inc., KY).

### Image Acquisition

Time-lapse Z-stacks were acquired using a Zeiss Axio Observer Z1 automated microscope (Carl Zeiss, Jena, Germany), with an Axioplan 20X (0.8NA) and 1.25 **μ**m Z steps. Images of the 53BP1-mCherry and H2B-GFP channels were acquired every 15 min for 24 hours prior to irradiation and 24 hours post-irradiation. The time interval was shortened to 5 min for the first hour post-irradiation, in order to capture fast foci events occurring early after exposure. We used the H2B-GFP channel for cell segmentation. Fig 1A–1C illustrate the imaging protocol and cell segmentation.

### Image Processing

All image processing were performed using CellAnimation [15], Fiji [16] and custom apps developed in MATLAB (Mathworks, MA) and Python. Maximum intensity projection (MIP) of Z-stacks was performed to visualize all foci and nuclei within one single 2D image. We used the trainable Weka segmentation plugin in Fiji to develop a classifier which assigned pixels to either foreground or background pixels. To segment nuclei clusters into individual nuclei we used a custom convexity restricted watershed. This was done by combining a standard distance watershed with an algorithm which excluded shapes having only convex angles from segmentation [17]. This was based on the observation that single nuclei are convex shapes while nuclei conglomerates exhibit concave angles. To eliminate non-significant concavities from shape contours we used a Douglas-Peucker line simplification algorithm[18]. This led to very accurate identification of individual cells (Fig 1C). Segmented cells were then automatically tracked using a custom pipeline in CellAnimation (Fig 1D). The pipeline detects mitotic events and assigns ancestry information. For these movies cells up to four generations of cells were detected (Fig 1E and 1F). For each time-lapse series the software generates two csv files. The tracking file lists the track ID and shape features (centroid position, area, perimeter, etc.) of every nucleus in the movie. The ancestry file lists the detected parent ID, start time and split time for every cell. In addition to the csv files, a set of images is produced which overlays the track IDs and cell generation on the original images. These images can be used for manual validation of the automatic tracking. We used the manual tracking review module of CellAnimation to correct erroneous mitotic events. To detect individual foci we developed a custom module for CellAnimation which uses the H-dome transform. This method has been shown to perform well for subcellular object detection [19].

### Data Processing

Cell and foci numerical features extracted from the time-lapse movies were processed using R (Team 2006) and RStudio (RStudio, MA). Foci classes were assigned based on their total area and mean fluorescence intensity. We used a Gaussian mixture model applied to a random subsample of 10,000 data points to determine the classes. To test the stability of the classes we applied the Gaussian mixture algorithm 10 times to different random subsets of 10,000 points each with a maximum of five clusters allowed. The algorithm consistently selected four classes as the optimum number of classes for the data with class boundaries similar between runs. To determine the conversion rates between different foci classes we used linear programming techniques. In addition to the four classes determined automatically we added a class 0 to account for protein which is not part of a focus (total intensity of protein not in foci). Based on mass balance principles the following equality applies for any foci class *k*:

$$I_{k}\left(t\right)=I_{k}\left(t-\delta t\right)+P_{k}-R_{k}$$ (1)

,where *I*_*k*_ (*t*) is the total 53BP1 intensity in class *k* at time step *t* and *I*_*k*_ (*t* − *δt*) is the total class *k* intensity at the previous step. *P*_*k*_ is the total amount of protein from foci in other classes being converted to class *k* in the time interval *δt*:

$$P_{k}=\sum_{j\neq k}C_{jk}I_{j}\left(t-\delta t\right)$$ (2)

,where *C*_*jk*_ is the conversion rate from class *j* to class *k* at time *t* − *δt*. *R*_*k*_ is the total amount of protein converted away from class *k* to foci in any other class:

$$R_{k}=\sum_{j\neq k}C_{kj}I_{k}\left(t-\delta t\right)$$ (3)

We set the objective function to be minimized to the sum of the conversion rates ∑_*j* ≠ *k*_ *C*_*kj*_.

## Results

### High-content analysis of live cell response to ionizing radiation at the population level

**Fig 2:**
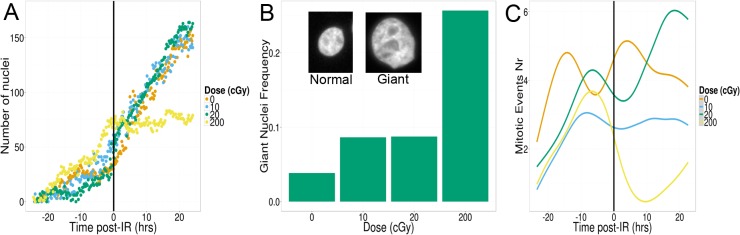
Radiation effects on population growth. (A) Radiation effect on numbers of nuclei. 10 and 20 cGy doses have a negligible effect on population growth. In contrast the 200 cGy dose results in growth arrest for 24 hours after irradiation. (B) Growth arrest is accompanied by an increase in the population of cells with giant nuclei (normal versus giant nucleus shown in inset) most likely due to a protective repair mechanism resulting in DNA duplication without mitosis. (C) Mitosis is impaired for cells receiving the 200 cGy dose with cells entering a period of mitotic arrest for as long as 10 hours post-IR. In contrast, cell division is not significantly affected by low doses of radiation.

The ability to monitor large populations of cells over days and to quantify individual cellular events allowed us to test well-established concepts about the response of human cells to ionizing radiation. Fig 2 illustrates the population response of MCF10A human mammary epithelial cells exposed to X-rays. Approximately 150 cells for each dose were tracked automatically, leading to the follow-up of as many as three cell generation within the 48 hours time-lapse. Any errors in mitotic event detection were corrected using the track review GUI of CellAnimation (see Material and Methods). Cells were exposed to radiation 24 hours after time-lapse recording started (Fig 2A, vertical black bar, time 0 hr). Overall cell growth was not affected by doses up to 0.2 Gy. In contrast, a higher dose (2 Gy) led to a complete arrest that lasted ~40 hours. Because we track individual cells, individual events such as cell death and mitotic events were also recorded. Cell death was identified as an event where a cell was present in the middle of the field of view and absent in the next time frame (cells close to the edge and disappearing were attributed to a cell moving out of the field of view). Mitotic events were defined by the generation of two new cells in the same vicinity, the loss of 53BP1-mCherry signal, and increased intensity of H2B-GFP signals. For doses less or equal than 0.2 Gy, no cell death was observed and mitotic events were not significantly different before and after exposure (Fig 2C). In contrast, at high dose (2 Gy), mitotic events were almost entirely absent within 20 hours following exposure. Interestingly, no significant cell death was recorded for 2 Gy, suggesting the cell number stall in Fig 2A is primarily the result of growth arrest and not cell death. This is corroborated by an increase in the number of cells with giant nuclei which represent cells that are duplicating their DNA without dividing. We defined a giant nucleus as a nucleus whose size is larger than the mean plus twice the standard deviation of the size of control cells.

**Fig 3:**
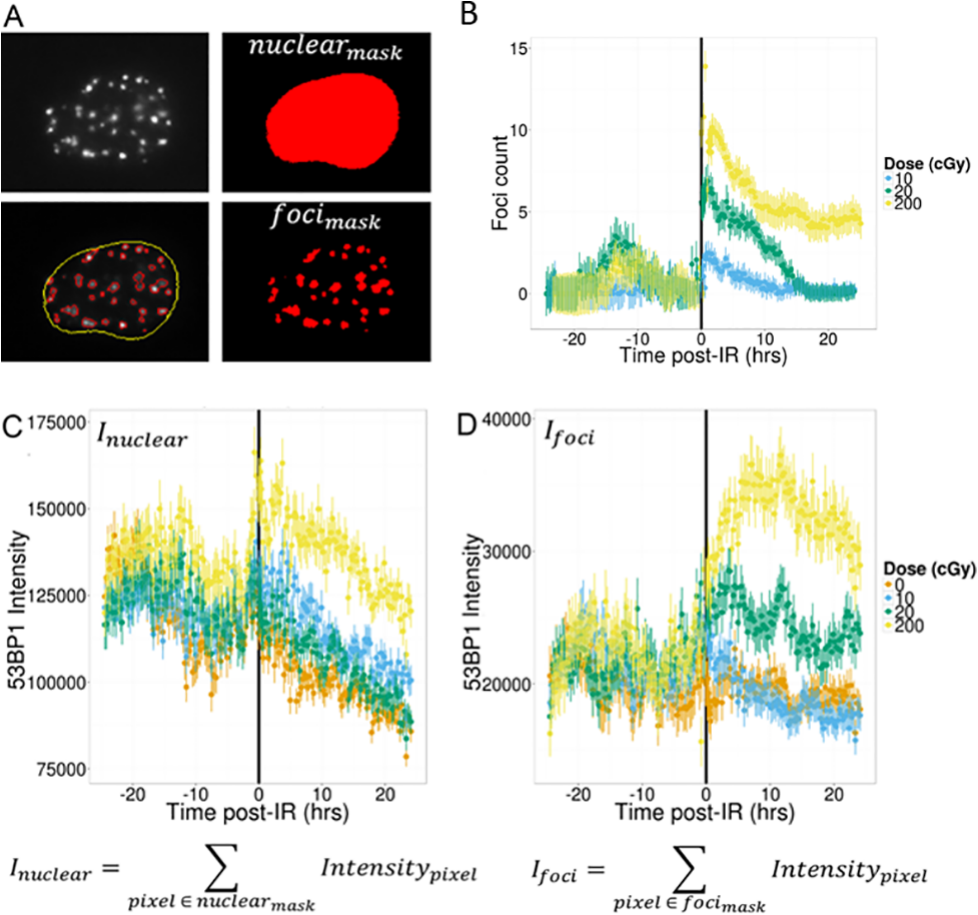
53BP1 Protein Dynamics. (A) This panel illustrates the various computations performed regarding RIF number and protein relocalization. We compute total protein intensity as the sum of pixel intensities in the entire nuclear area (*I*_*nuclear*_) for all nuclei present in each time frame. RIF are detected using H-dome transform (see Material and Methods) and are depicted in the image as red mask. The total amount of proteins recruited in all RIF is computed as the total foci intensity in these masks for each time frame and normalized to the number of nuclei (*I*_*foci*_). (B) RIF numbers are corrected by subtracting number of spontaneous foci measured in the 0 cGy sample (see text for details). (C) Total 53BP1 nuclear intensity suggests that cells exposed to 200 cGy produce additional proteins following ionizing radiation as its curves depart from the other doses. (D) If we quantify the change in protein that is present in foci (excluding background protein) we observe a marked increase for both 200 and 20 cGy but not for the low dose when compared to control.

To get a global view of the DNA damage response in cells, we first averaged foci information on a per field of view basis normalizing by the number of nuclei in the field. Fig 3A illustrates the automatic nuclear segmentation and RIF detection (see Material and Methods for more details). RIF numbers were high for all specimens at the beginning of the experiment and decreased as cells settled in their new environment. Using the control RIF number over this 48 hour time course, we modeled this decrease by an exponential decay. This idealized curve was subtracted to the number of RIF for all doses (Fig 3B). Such approach has the advantage of not adding supplemental noise that would occur if directly subtracting the sham RIF curve. The resulting RIF time dependence (Fig 3B) follows the classic dome shape we previously reported for these cells [4]. More specifically, we measure a slower repair at high dose: the average half-life for RIF resolution measured by exponential decay over the first 20 hours post-IR was ~7 hours for 2 Gy against ~4 hours for 0.1 Gy. Surprisingly, the total amount of 53BP1 intensity per cell does not remain constant as one would expect. Instead, we observe a large and almost immediate increase following 2 Gy compared to all other doses (Fig 3C). This suggests either rapid production of newly synthesized 53BP1 or immediate inhibition of 53BP1 degradation following large doses of ionizing radiation. When measuring the total amount of 53BP1 proteins recruited inside RIF (Fig 3D), we notice that it is significantly larger than for spontaneous foci when the dose is greater or equal to 0.2 Gy. In contrast, 0.1 Gy produces RIF that are similar to spontaneous foci. In addition, protein recruitment inside high dose RIF is delayed compared to RIF resolution with peaks reached only 10 hours following 2 Gy (Fig 3D–note that 0.2 Gy does not show a clear peak with an increased intensity plateauing between 2 and 10 hour post-IR). Finally, both RIF number and RIF intensity peak ratio between 0.2 Gy and 2 Gy are much less than the 10 fold dose ratio, suggesting saturation in the detection process.

### Foci classes, protein recruitment and Foci merging

**Fig 4:**
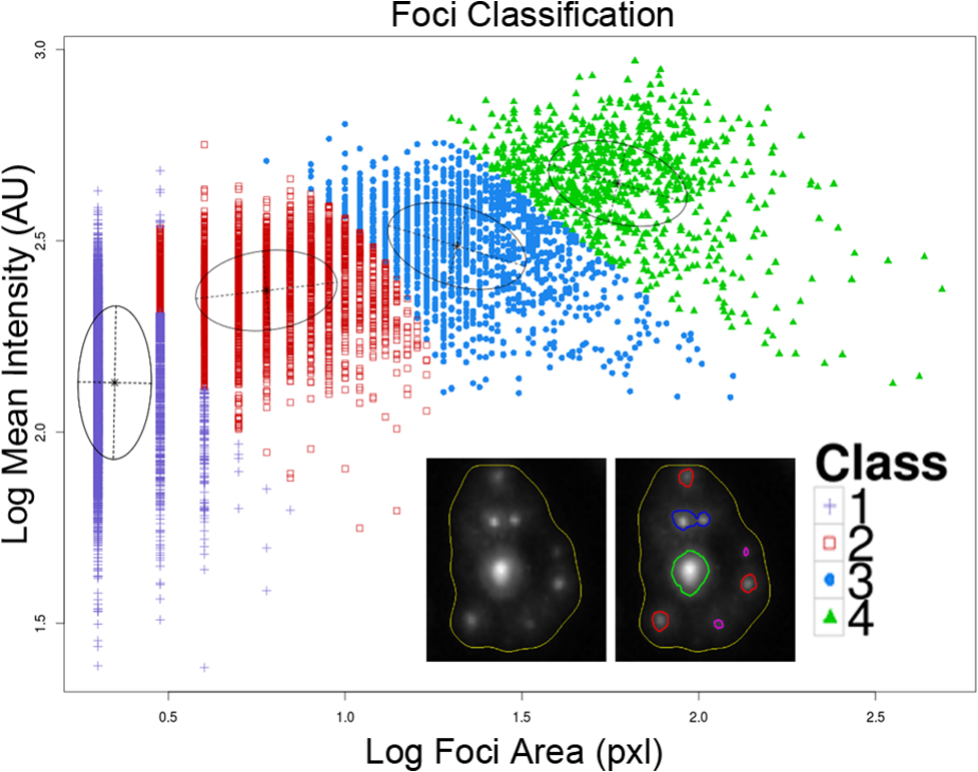
Foci classes. Scatterplot of foci mean intensity versus foci area. A Gaussian mixture model applied to these two parameters is used to split foci into four classes. Class 1 contains small area and lower intensity signal, Class 2 represents bright small clusters, class 3 medium size clusters and class 4 large clusters. Inset cell illustrates all four foci types. 1 pixel = 0.1 square microns.

**Fig 5:**
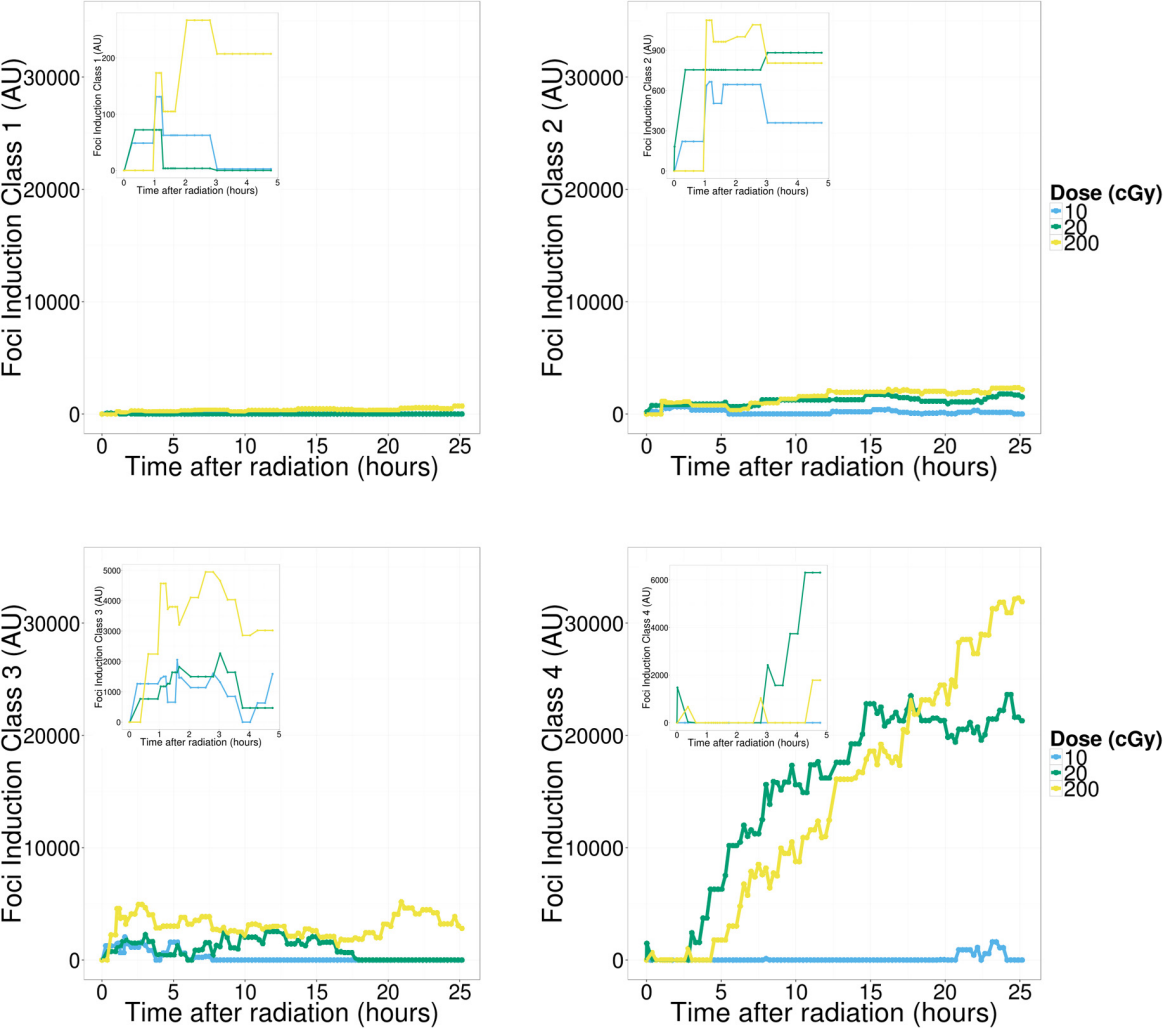
Foci induction. Cumulative count of foci being induced from background protein in classes 1,2, 3 and 4 segregated by dose. Counts have been normalized by subtracting the sham dose counts and setting any value below sham to zero. We see the strongest radiation induced induction in class 4 (large clusters). There seems to be no significant response at 10 cGy and the induction at 20 cGy tapers off to control levels after about 15 hours. In contrast induction at 200 cGy does not return to control levels even after 25 hours. Induction of class 4 foci also exhibits a dose dependent delay where cells are seen to respond faster to the lower dose.

**Fig 6:**
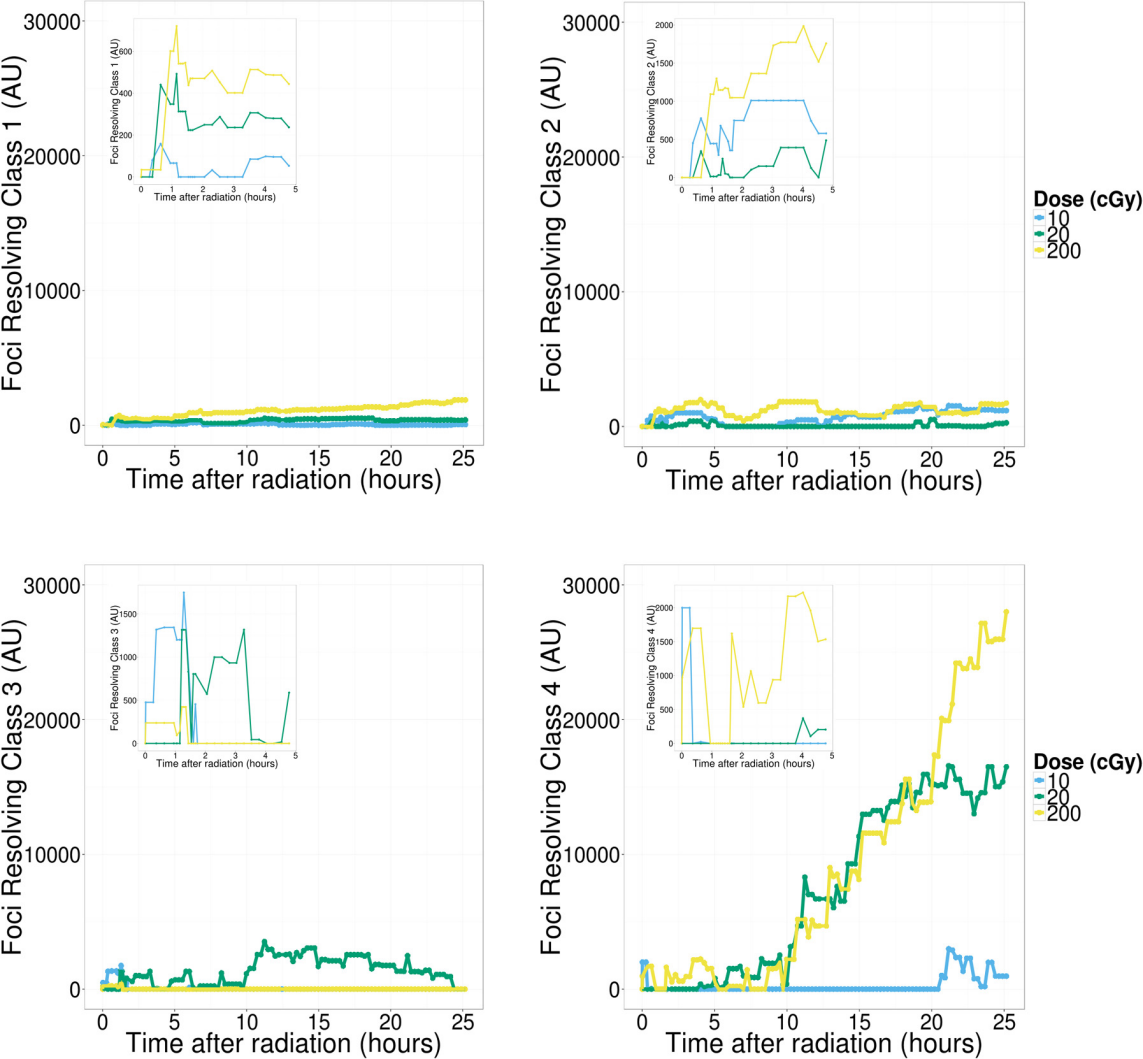
Foci resolving. Cumulative count for foci resolving to background protein. Similar to induction the highest amount of protein is returned to background from the largest clusters (class 4). Repair in cells exposed to 20 cGy levels off after about 20 hours, while repair in the 200 cGy dose continues unabated for the duration. In class 3 foci we observe a radiation response only for the 10 cGy dose. Low levels of radiation induced repair are also observed in the small clusters (class 1 and 2) but the majority of foci resolving to background occurs in the largest clusters (class 4).

**Fig 7:**
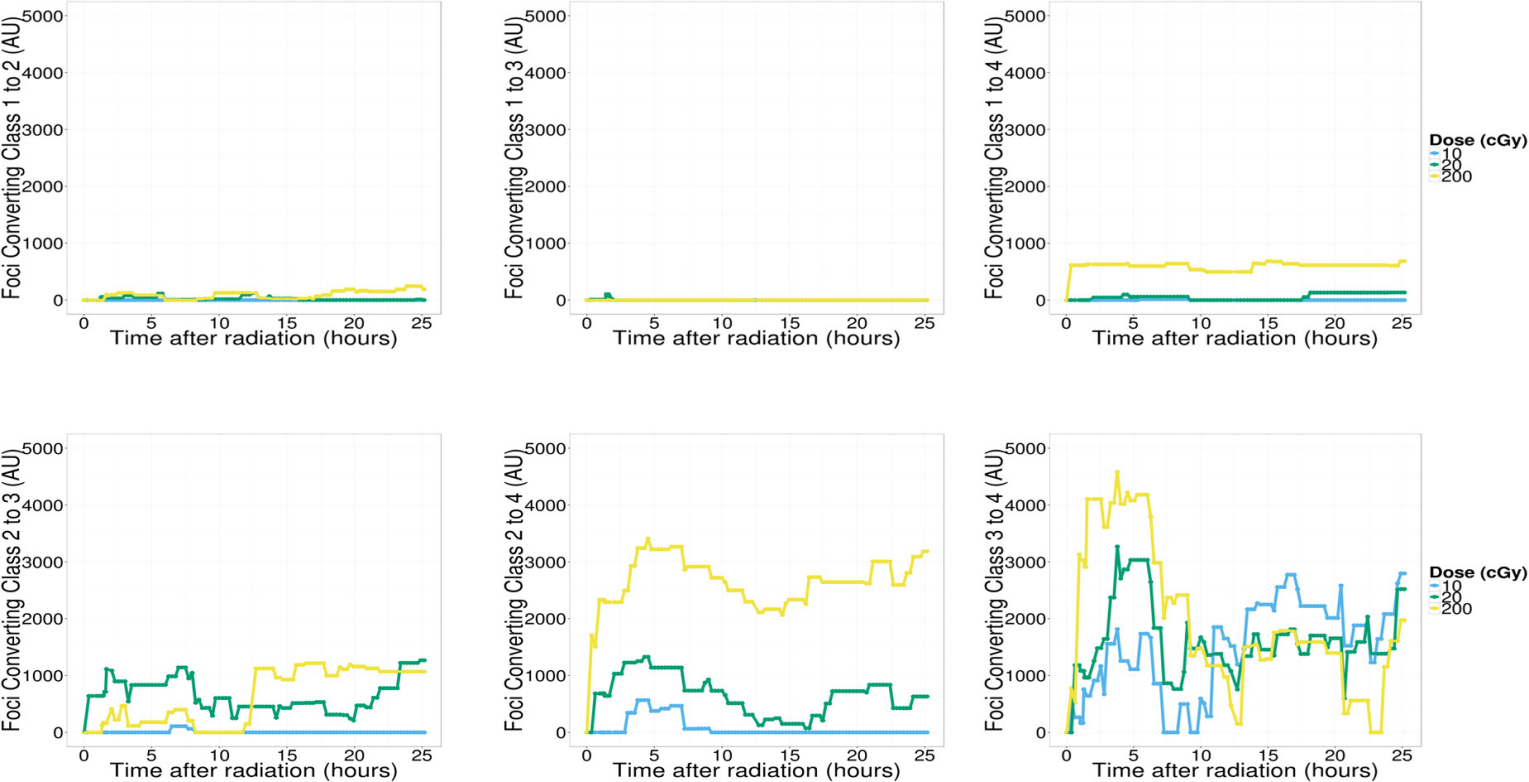
Foci merging. Conversion to larger clusters. We observe the clearest dose-dependent radiation response in the conversion of classes 1 and 2 to class 4 which is the class where the majority of foci resolving occurs. We also observe a complete lack of response in conversion from classes 1 and 2 to class 3. This is indicative of a clustering process where smaller clusters move into the largest clusters in contrast to a growth process where clusters enlarge and the growth is reflected in an increase in the total protein of clusters of next highest volume.

Results from the previous section suggest active 53BP1 protein movement in and out of RIF for hours following exposure to ionizing radiation and such movement is probably dose dependent. In order to better characterize the repair process we introduce an imaging classifier based on RIF size and mean pixel intensity. This is done by separating foci into different classes, using a Gaussian mixture model based on size and intensity features of a randomly sampled subset of 10,000 foci (Fig 4). This process is repeated ten times to ensure cluster boundaries are stable, leading to four classes of foci going from small and dim (class 1) to very large and bright foci (class 4). The time responses for individual foci classes (data not shown) show very distinct kinetic and we hypothesize that the DNA damage response leads to a dynamic progression between foci classes. We tested this hypothesis by using linear programming techniques (see Material and Methods), to compute conversion rates between the different foci classes and background protein. We defined any conversion from any other foci class to the background protein class 0 as foci being resolved. In contrast, we defined conversion from class 0 to any other class as foci induction. Graphs depicted in Figs 5 – 7 plot the total amount of proteins (pixel intensity) going in and out of the various foci classes.

Foci induction is depicted in Fig 5. Surprisingly, the majority of protein recruitment goes towards the largest foci (i.e. class 4). Note that even though there are more foci created for lower classes, it involves a lot less protein making their graphs negligible in comparison to higher classes. For all doses considered, foci induction for smaller classes are characterized by a much faster kinetic. In contrast, class 4 induction is significant for doses greater or equal to 0.2 Gy. There is a 4 to 5 hours post-IR delay for the induction of class 4. The 0.2 Gy dose elicits an induction response which returns to control levels after ~15 hours. Cells exposed to 2 Gy have an induction that continue unabated for the entire duration of the time-lapse. Similarly, the largest movement of protein for foci resolution is from class 4 (Fig 6) with a significant delay that is inversely proportional to dose. More specifically, the time delay component is ~10 hours, roughly double the amount of time that the induction component is delayed for the highest dose. This five hour additional delay probably reflects the repair process in these large structures. The slope of the cumulative resolving curve for 2 Gy and 0.2 Gy is equal in the period of maximum response, possibly an indication that the resolving capacity is maximized. As with induction we observe a return to control levels for the 0.2 Gy dose after ~20 hours while the resolving response continues unabated for the 2 Gy dose.

We also monitor the conversion of protein from smaller foci classes to larger ones (Fig 7) a process which may be caused by foci growth or foci clustering. In the case of foci growth one would expect to see higher conversion rates between neighboring classes such as class 1 converting to class 2, class 2 to class 3 and so on. Instead what we observe is that the best dose dependent radiation response occurs in conversion of class 1 and class 2 to class 4. In contrast conversion of class 1 to 2 and 1 to 3 shows no radiation response and the conversion of class 2 to 3 does not seem to be dose dependent. This indicates that the conversion of smaller foci to larger ones occurs through a process of clustering of individual foci into a larger one as opposed to gradual increase in size of an individual focus. Radiation dependent conversion does occur from class 3 to class 4 for the first 10 hours indicating an increase in size from medium to large clusters. The clustering response occurs much faster following radiation than the delayed responses of induction and resolving. The time order of these events is clustering, followed by induction, followed by resolving to background. If we assume that most of the resolved protein indicates repair we can conclude that most repair occurs inside large clusters.

### DNA repair domains

**Fig 8:**
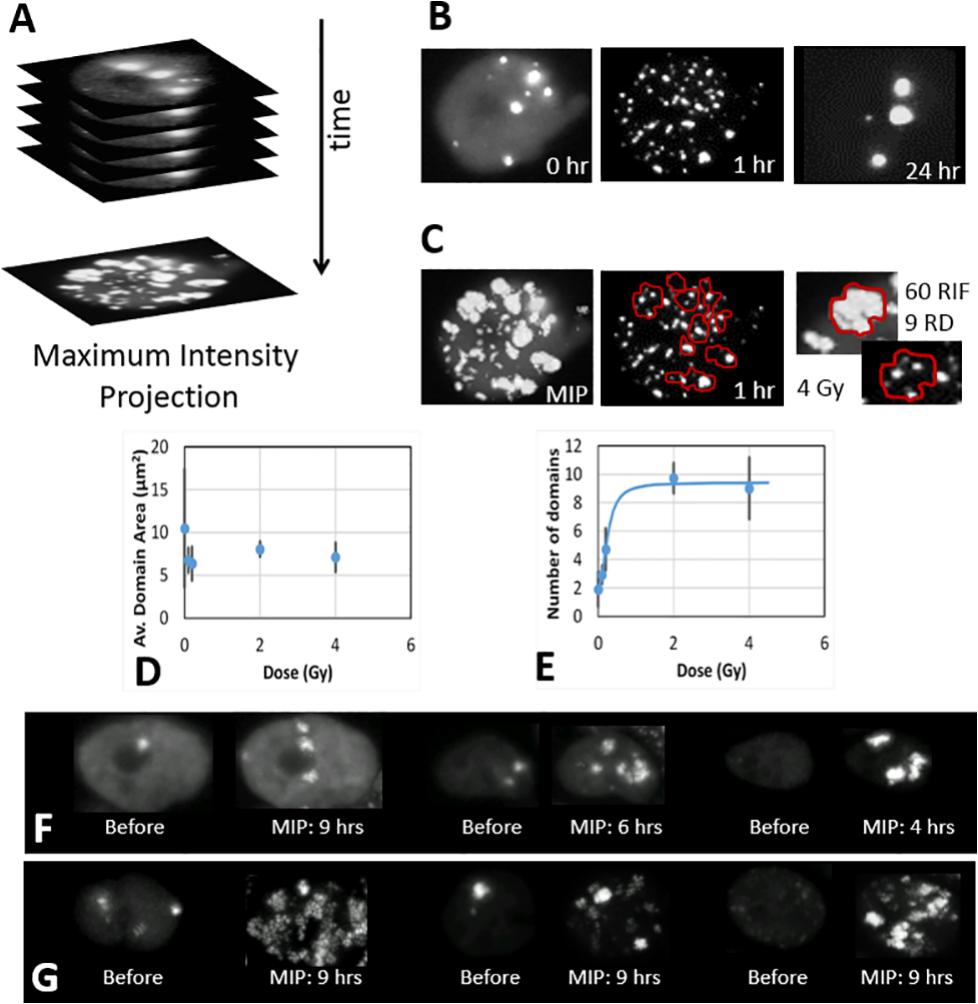
Human breast cells MCF10A have a limited number of repair nuclear domains. A) Movie of a registered nucleus over a 24 hour time course following 4 Gy of X-ray is projected across all time frames into one single frame using maximum intensity projection (MIP) transformation. B) Various snapshots of the movie. Note that large and bright foci are present before IR and are recovered after repair is complete. C) MIP of the movie reveals very clear nuclear domains patterns, referred as Repair Domains (RD). RIF stay within these domains (RD contours shown in red overlaying the 1 hr time frame). In this example, this cell processes ~60 RIF within 9 RD. D) The average domain area remains relatively constant regardless of dose at about 7.5 **μ**m^2^. E) As dose increases, the number of domains reaches a constant value, suggesting human breast cells MCF10A have a total of 9.3 RD/cell on average. F-G) Representative images of cells just before being exposed to ionizing radiation and for a duration of 4 to 9 hours following 10 cGy (F) and 200 cGy (G). Images before radiation show a projection combining only 45 minutes (3 time frames) whereas projection for irradiated cells combine up to 36 frames (~9 hours).

The previous section suggests that DNA repair mechanisms for MCF10A involve RIF merging. Since all cells have been tracked individually, we can crop sub-regions by centering the time-lapses on each individual recorded nuclear positions. To further eliminate small lateral or rotational movements of the centered nuclei, we use elastic registration algorithms from OpenCV (http://opencv.org). Many cells are lost in this process because they either leave the field of view during the time-lapse or they get deformed as they move and roll over time. Movies of single cells exposed to various doses of ionizing radiation have been generated this way and are available in supplemental data. In order to test if foci reside in preferential regions of the nucleus, we can then collapse a set of time frames (4 to 24 hours post-IR) following exposure to ionizing radiation into one single frame using maximum intensity projection algorithm (Fig 8A). Fig 8B shows key time frames of a single cell after registration after a 4 Gy exposure. One can observe 9 repair domains clearly visible in the MIP image and how individual foci are constrained within these domains (Fig 8C). It is interesting to note that the average area of repair domains remains constant at ~7.5 **μ**m^2^ regardless of dose (Fig 8D). The number of repair domains increases from a low of 2 for control cells reaching its asymptotic plateau of 9.3 passed 1 Gy (Fig 8E). Fig 8F and 8G illustrate domains observed with MIP of registered time-lapse for 0.1 and 2 Gy (Fig 8F and 8G respectively).

**Fig 9:**
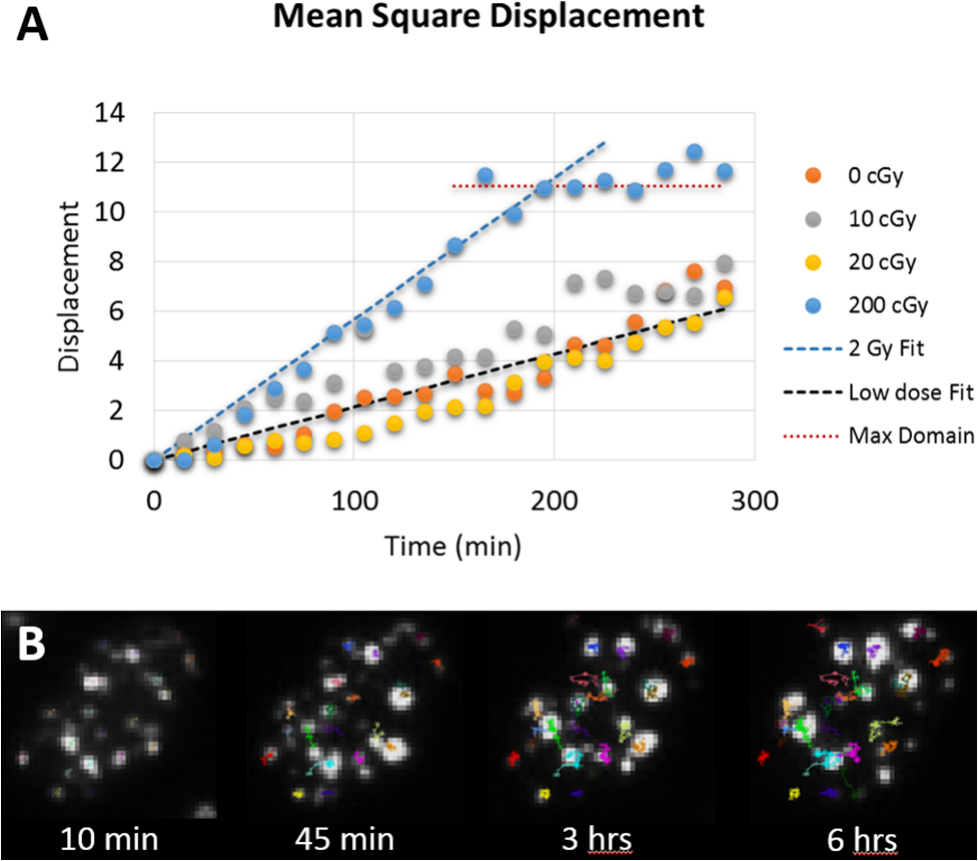
RIF mean square displacement. A) Plot showing the mean square displacement of foci as a function of time post-IR. Control (0 cGy) show the movement of spontaneous foci measured in non-exposed wells for the same time points. 26, 22, 45 and 61 foci were tracked manually using ImageJ plugin for 0, 10, 20 and 200 cGy respectively. B) Snapshot of foci tracking algorithm for 2 Gy response, showing each individual tracks with various colors.

In order to better characterize foci movement within these domains, one can track the mean square displacement of individual foci after exposure to ionizing radiation in registered nuclei. Foci tracking is challenging and for better accuracy, this was done by hand for 20 to 60 RIF per dose point. The mean square displacement is displayed as a function of time post-IR showing a clear linear dependence with time, which suggests RIF move randomly following classic diffusion motion (Fig 9A). In addition, the diffusion rate is dose dependent with 0.014 **μ**m^2^/min after 2 Gy against 0.005 **μ**m^2^/min for any doses less or equal than 0.2 Gy. Interestingly, the 2 Gy dose clearly highlights the domain limit with a movement constrained within an 11 **μ**m^2^ area. This is larger than what was estimated by MIP probably reflecting the fact that individual RIF can explore further than the apparent domains, as one can observe in individual foci tracks (Fig 9B).

## Discussion

We [4, 20] and others [21] have previously shown strong non-linear RIF yields in non-malignant human breast cell line MCF10A exposed to ionizing radiation. In our previous work [4] we also showed that the lower number of RIF/Gy at higher doses was accompanied with a faster RIF induction and slower RIF resolution. Live cell imaging has been used to address these kinds of questions before [22, 23] but the lack of sophisticated imaging tools makes analysis limited to very few cells. In this work, we introduce a suite of algorithms that allows quantification of hundreds of living cells over a 48 hour time-course in large fields of view using low magnification objective. Cells are exposed to focused 40 kV X-ray millibeam, allowing irradiation while cells are being imaged (manuscript in preparation). By being able to quantify in an unbiased manner hundreds of cells and millions of foci, a more accurate picture of the DNA repair response of human cells appears, indicating distinct responses between low and high doses of radiation.

Because time frames are acquired at a high frequency, and individual cells are tracked continuously, our approach allows us to quantify dynamically mitotic events and cell death in ways that could not be done before, allowing us to revisit “text book principles” for radiation biology. With classic clonogenic assays or cell death dyes, one can only measure cell death at a fixed time post-irradiation. In contrast with our approach, all cell deaths are captured. It is therefore interesting to note that in contrast to the classic dogma of radiation induced cell death, negligible levels of cell death is observed for doses as high as 200 cGy. Further investigation has shown that this is true for duration as long as 48 hours following 200 cGy (data not shown). Instead of cell death, many MCF10A become extremely large, suggesting some form of senescence for immortalized epithelial cells. On the other hand, as expected, 200 cGy leads to an immediate growth arrest with mitotic index dropping to zero. MCF10A show no cell-cycle arrest for doses as high as 20 cGy, suggesting such doses do not elicit all cell-cycle checkpoints. 20 cGy should elicit only 7 DSB. Therefore our results are in good agreement with previous studies suggesting a cell require at least 10 to 20 DSB before it activates its cell cycle checkpoint [24].

Regarding dose and temporal dependence, we recapitulate previous results [4] showing the number of RIF saturates with increasing doses while RIF appear faster. Saturation is not as pronounced if we report the total amount of 53BP1 recruited within all RIF in the nucleus instead of the number of RIF. This confirms our recent work on high-LET RIF showing total RIF intensity is a better biodosimeter than RIF number [13]. The most unexpected result however is the fact that 53BP1-mCherrry overall nuclear intensity goes up as fast as 5 min after exposure to 2 Gy, suggesting production of new protein or inhibition of proteolytic activity. This is not observed for lower doses such as 0.2 Gy suggesting a dose threshold is necessary to activate this response. More work is needed to understand the mechanism at play here and we can only speculate that it reflects a feedback to better handle the larger amount of DNA damage. On the other hand, protein recruitment within a focus shows subtle differences for much lower doses with the total foci intensity increasing with dose, for doses larger or equal to 0.2 Gy. This additional protein recruitment within foci occurs over a long period of time with a maximum reached 10 hours post-IR instead of the classic 30 min peak for RIF resolution. This delay in kinetic is probably reflecting RIF clustering as discussed in the next section. This increase is not notable for doses lower or equal to 0.1Gy and in general our results on 53BP1 protein levels suggest doses less than 0.1 Gy elicit RIF that cannot be distinguished from spontaneous foci. In contrast, higher doses elicit RIF and protein changes that may not be linear with dose and whose properties are unique to ionizing radiation. This is an important finding as 0.1 Gy is considered to be the threshold under which one considers the radiation dose to be low, based on the fact that no significant cancer risk has been observed below 0.15 Sv [25]. The fact that DNA repair observed via RIF formation cannot be distinguished from repair under physiological conditions for doses less or equal to 0.1 Gy seems in good agreement here.

Many studies have suggested in the past that DSB repair takes place at fixed positions in the nucleus near the site of damage [8, 9]. More recent studies revealed that RIF can also explore the nuclear space during repair [5–7, 11, 26–28]]. Most RIF motions are believed to be limited, constrained and sub-diffusive [6, 9, 28, 29], but no clear imaging evidence has been put forward to characterize these repair domains. We previously reviewed extensively this issue [12], showing how the various possible mechanisms for DSB detection and protein recruitment to the damaged site, and the movement of damaged sites are all factors complicating the interpretation of RIF kinetic. In the work presented here, we can separate the impact of protein recruitment to the damaged site with the movement of the DSB itself by classifying RIF into four sub-category of foci defined by their size and intensity (largest and brightest foci define the class number 4). In addition, a mathematical approach is proposed to compute the most likely rate of conversion from one foci class to another class. Such approach allows us to conclude that the main mechanism by which RIF are formed following high doses of IR is via RIF clustering (small classes converting into larger ones). In contrast, low dose response is characterized by direct protein recruitment to the individual foci classes.

Time projections of time-lapse of individual nuclei give us more clue as to how clustering is occurring in the nucleus. In our work, we show that RIF movement is constrained within finite confined nuclear regions. The actual size of these repair domain remain uncertain as tracking of individual foci suggest that RIF move randomly via diffusive processes in domain as large as ~11 **μ**m^2^, whereas time-lapse projections suggest domain sizes of 7.5 **μ**m^2^. However, individual foci tracking measurements may be biased towards larger distances because they exclude merging foci. In any case, these results clearly identify preferential regions of the nucleus where repair must take place. We previously showed that RIF appear preferentially in low DNA density regions for human cells [3] and we later confirmed movement of RIF from heterochromatic to euchromatic regions for homologous recombination in drosophila [5]. More recently, we developed a computer model of artificial human cells showing that repair domains can explain and predict the higher cell killing efficiency for doses larger than 1 Gy [13]. This is because allowing DSB to move within constrained volume increases the likelihood of DSB proximity, DSB misrepair and eventually cell death. In this model, the domain size was estimated to occupy 6% of the nucleus [13]. The cells used here have an average nuclear surface of ~150 **μ**m^2^, therefore our theoretical model suggests a domain size of ~9 **μ**m^2^ which is in good agreement. It is important to note that these experimental results are for cycling cells, which have a large contributions of G2 cells. In contrast, our theoretical predictions were done on confluent cells, with 95% growth arrest and much smaller nuclear surfaces. In addition, our previous work did not take into account the different diffusion rates of foci as a function of dose, clearly indicating that more work is required.

The societal impact of this work is important. Current risk management for exposure to ionizing radiation assumes that cancer risk is linear from Gy to **μ**Gy [30]. This is referred to as the linear-no-threshold (LNT) model. In contrast, our high-throughput image processing pipeline show that high doses of radiation elicit a variety of biological phenomena in the DNA repair response that are not observed or that are extremely rare for low doses. It is therefore difficult to justify the LNT regulatory approach based on DNA damage alone, which has assumed for the longest time that since the amount of DNA damage is proportional to dose, risk should also be linear. This has of course been supported by epidemiologic studies from A-bomb survivors [30],but interpretation in the low dose range has remained a highly controversial topic [31] and inconclusive below 15 cGy [25]. However, our results do not prove or disprove the LNT, it just removes DNA damage as a potential mechanism explaining LNT. We cannot conclude either that there is no risk from ionizing radiation at very low doses. We have in fact recently showed that other events, not directly related to DNA damage, can elicit global and systemic responses in a full organism {tang,2013}. These systemic changes can either enhance tumor progression in certain genotypic hosts [32] while inhibit cancer progression in other genotypes [33]. Some questions remain to be answered such as the amount of variation for RIF clustering and repair domains in the population. Can these phenomena explain specific organ sensitivity and individual sensitivity to ionizing radiation?

## Supporting Information

S1 Movie First cell used in Fig 8F exposed to 10 cGy. Movie has 46 frames at 8 frames/sec. The first 4 frames show the cell every 15 min before radiation. Frame 5 and forward show the cells post exposure to 10 cGy. Frame 5–13 have 5 min intervals with frame 13 at time 45 min post-IR. Frames 14–46 have 15 min interval, reaching 9 hours post-IR at frame 46. Images “before IR” shown in Fig 8F is a maximum project of frames 1–3. MIP in Fig 8F are based on maximum projection of frames spaced every 15 min (i.e. frame 7, 10, 13, and 14–46).
(AVI)

S2 Movie Second cell used in Fig 8F exposed to 10 cGy. Movie has 46 frames at 8 frames/sec. The first 4 frames show the cell every 15 min before radiation. Frame 5 and forward show the cells post exposure to 10 cGy. Frame 5–13 have 5 min intervals with frame 13 at time 45 min post-IR. Frames 14–46 have 15 min interval, reaching 9 hours post-IR at frame 46. Images “before IR” shown in Fig 8F is a maximum project of frames 1–3. MIP in Fig 8F are based on maximum projection of frames spaced every 15 min (i.e. frame 7, 10, 13, and 14–46).
(AVI)

S3 Movie Third cell used in Fig 8F exposed to 10 cGy. Movie has 46 frames at 8 frames/sec. The first 4 frames show the cell every 15 min before radiation. Frame 5 and forward show the cells post exposure to 10 cGy. Frame 5–13 have 5 min intervals with frame 13 at time 45 min post-IR. Frames 14–46 have 15 min interval, reaching 9 hours post-IR at frame 46. Images “before IR” shown in Fig 8F is a maximum project of frames 1–3. MIP in Fig 8F are based on maximum projection of frames spaced every 15 min (i.e. frame 7, 10, 13, and 14–46).
(AVI)

S4 Movie First cell used in Fig 8G exposed to 200 cGy. Movie has 46 frames at 8 frames/sec. The first 4 frames show the cell every 15 min before radiation. Frame 5 and forward show the cells post exposure to 200 cGy. Frame 5–13 have 5 min intervals with frame 13 at time 45 min post-IR. Frames 14–46 have 15 min interval, reaching 9 hours post-IR at frame 46. Images “before IR” shown in Fig 8G is a maximum project of frames 1–3. MIP in Fig 8G are based on maximum projection of frames spaced every 15 min (i.e. frame 7, 10, 13, and 14–46).
(AVI)

S5 Movie Second cell used in Fig 8G exposed to 200 cGy. Movie has 46 frames at 8 frames/sec. The first 4 frames show the cell every 15 min before radiation. Frame 5 and forward show the cells post exposure to 200 cGy. Frame 5–13 have 5 min intervals with frame 13 at time 45 min post-IR. Frames 14–46 have 15 min interval, reaching 9 hours post-IR at frame 46. Images “before IR” shown in Fig 8G is a maximum project of frames 1–3. MIP in Fig 8G are based on maximum projection of frames spaced every 15 min (i.e. frame 7, 10, 13, and 14–46).
(AVI)

S6 Movie Third cell used in Fig 8G exposed to 200 cGy. Movie has 46 frames at 8 frames/sec. The first 4 frames show the cell every 15 min before radiation. Frame 5 and forward show the cells post exposure to 200 cGy. Frame 5–13 have 5 min intervals with frame 13 at time 45 min post-IR. Frames 14–46 have 15 min interval, reaching 9 hours post-IR at frame 46. Images “before IR” shown in Fig 8G is a maximum project of frames 1–3. MIP in Fig 8G are based on maximum projection of frames spaced every 15 min (i.e. frame 7, 10, 13, and 14–46).
(AVI)

S7 Movie Shows one full movie with individual tracking of foci in one cell exposed to 200 cGy. (7 frames/sec, with the same frame scheme as described above, except frame 1 is the first frame post-IR). The duration of the movie is much longer than what was used to generate foci diffusion computation in Fig 9A, which stopped after 6 hours. Snapshots of this movie were used for Fig 9B.
(AVI)

S8 Movie Registered movie from cell exposed to 4 Gy, used to generate Fig 8ABC. 223 frames at 8 frames/sec Frames 1–26 is before IR with 15 min interval, covering 6.5 hours before radiation. Frame 27 is 5 min after IR and frames are acquired every 5 min until reaching 1.5 hour post-IR at frame 44. Frames are then acquired every 15 min for a total movie length of 30 hours post-IR. MIP in Fig 8C was done for the first 24 hours post-IR, with only using frames spaced every 15 min, starting at frame 35.
(AVI)
